# Advances in Perovskite Quantum Dots and Their Devices: A New Open Special Issue in *Materials*

**DOI:** 10.3390/ma15186232

**Published:** 2022-09-08

**Authors:** Shuangyi Zhao, Zhigang Zang

**Affiliations:** Key Laboratory of Optoelectronic Technology & Systems (Ministry of Education), Chongqing University, Chongqing 400044, China

‘*Advances in Perovskite Quantum Dots and Their Devices’* is a new open Special Issue of *Materials* in which original and review papers on the novel findings of synthesis, deep understandings of properties, and advanced potentials of applications in perovskite quantum dots are reported and published.

Owing to their prominent properties including tunable bandgaps, strong absorption, efficient emission, and long carrier diffusion lengths, perovskites composed of metal-halide have rapidly emerged as materials with great potential in the last decade. Thus, their wide applications in solar cells, light-emitting diodes, lasers, photodetectors, X-ray scintillators, and catalysis have attracted the attention of researchers and captivated the broad scientific community.

Furthermore, with the adaption of bulk perovskites to nanoscale ones, quantum dots exhibit a vast size-dependent optoelectronic performance, suggesting promising commercialization ability [1]. For instance, the tunable emissive wavelengths, narrow luminescence and high photoluminescent quantum yield up to ~100%—which are attributed to quantum confined effect and high exciton binding energies—make them excellent candidates in applications for displays with high color purity and broad color gamut, as well as illumination with controllable color temperatures. In addition, perovskite quantum dots are regarded as state-of-the-art materials for efficient solar cells and photodetectors because of their strong optical absorption, easy solution processability, and long carrier diffusion lengths [2]. Thus, it is of great significance to highlight and discuss the advances in the field of perovskite quantum dots.

The research interests of this Special Issue include, but are not limited to, the following: the exploration of preparation methods; the scientific understanding of structures and optoelectronic mechanisms; the optimization of properties; wide-ranging applications; and the fabrication and performance enhancement of devices based on perovskite quantum dots.
